# Fumigant activity and transcriptomic analysis of two plant essential oils against the tea green leafhopper, *Empoasca onukii* Matsuda

**DOI:** 10.3389/fphys.2023.1217608

**Published:** 2023-09-19

**Authors:** Weiwen Tan, Ni Zhang, Jinqiu Wang, Tianyi Pu, Jifeng Hu, Can Li, Yuehua Song

**Affiliations:** ^1^ School of Karst Science, Guizhou Normal University, Guiyang, China; ^2^ State Engineering Technology Institute for Karst Desertification Control, Guiyang, China; ^3^ Huaxi District Plant Protection Station of Guiyang City, Guiyang, China; ^4^ Guizhou Provincial Key Laboratory for Rare Animal and Economic Insect of the Mountainous Region, Guiyang University, Guiyang, China

**Keywords:** *Empoasca onukii*, plant essential oil, fumigant activity, transcriptome, detoxification genes

## Abstract

**Introduction:** The tea green leafhopper, *Empoasca* (*Matsumurasca*) *onukii* Matsuda, R., 1952 (Hemiptera: Cicadellidae), is currently one of the most devastating pests in the Chinese tea industry. The long-term use of chemical pesticides has a negative impact on human health, impeding the healthy and sustainable development of the tea industry in this region. Therefore, there is a need for non-chemical insecticides to control *E. onukii* in tea plants. The essential oils from plants have been identified for their potential insecticidal ability; however, there is a lack of knowledge regarding the effect of plant essential oils on *E. onukii* and its gene expression.

**Methods:** In order to address these knowledge gaps, the components of *Pogostemon cablin* and *Cinnamomum camphora* essential oils were analyzed in the present study using gas chromatography‐mass spectrometry. The fumigation toxicity of two essential oils on *E. onukii* was tested using sealed conical flasks. In addition, We performed comparative transcriptome analyses of *E. onukii* treated with or without *P. cablin* essential oil.

**Results:** The 36-h lethal concentration (LC_50_) values for *E. onukii* treated with *P. cablin* and *C. camphora* essential oils were 0.474 and 1.204 μL mL^−1^ respectively. Both essential oils exhibited the potential to control *E. onukii*, but the fumigation activity of *P. cablin* essential oil was more effective. A total of 2,309 differentially expressed genes were obtained by transcriptome sequencing of *E. onukii* treated with *P. cablin* essential oil.

**Conclusion:** Many of differentially expressed genes were found to contain detoxifification genes, indicating that these families may have played an important role when *E. onukii* was exposed to essential oil stress. We also found differential expression of genes related to redox-related gene families, suggesting the upregulation of genes associated with possible development of drug and stress resistance. This work offers new insights for the prevention and management of *E. onukii* in the future.

## 1 Introduction

*Empoasca* (*Matsumurasca*) *onukii* Matsuda, R., 1952 is an important agricultural pest that damages tea plants. By sucking on the young sections of tea plant tips, adults and nymphs deplete the chlorophyll and sap in buds and leaves. Females also place their eggs on the young tips, blocking the usual nutrient passage and causing cessation of bud and leaf growth. The leaf border and leaf tip turn reddish brown and wither when the damage is severe (Jin et al., 2012). Damaged tea leaves are fire-like and easily broken during processing, which negatively impacts tea quality and output and can result in yield reductions by 15%–20%, and up to 50% in extreme cases (Chen et al., 2019; Zhang, 2019; Zhang and Chen, 2015). These insects are small, tend to hide, have overlapping generations, and cause serious damage; hence, their control is difficult. In recent decades, chemical pesticides have been mainly relied on to prevent and control this pest; however, this is a high-cost method that induces strong resistance in the insects and produces pesticide residues that are harmful to human health. The improvement of pesticide residue limits in foreign countries has considerably hindered the export of tea products from China (Fan, 2021); hence, traditional chemical pesticide control can no longer meet current requirements.

Plant essential oils, also known as volatile plant secondary metabolites, are volatile oils with a significant aromatic component that gives aromatic plants a particular odor, flavor, or smell; they are commonly referred to as metabolic by-products of plants (Pavela, 2015). Plant essential oils have recently gained popularity due to their environmental friendliness and safety for non-target organisms when used to control food storage pests, phytophagous pests, and mosquitos (Filomeno et al., 2017; Muturi et al., 2017; Visakh et al., 2022). Fumigation or exposure to plant essential oils may have toxic effects on phytophagous insects; in addition, plant essential oils can affect the behavioral activities of insects, including egg laying, reproduction, feeding behavior, growth inhibition, etc. (Su et al., 2018; Ikbal and Pavela, 2019). The mechanism of action of various essential oils varies as a result of the complexity of the essential oil composition. One theory suggests that plant essential oils affect normal growth, development, metamorphosis, and reproduction by acting directly on the nervous system (acetylcholinesterase, ionotropic GABA receptors, and metabolic octopamine receptors) such as octopamine and tyramine receptors that regulate insect metabolism and behavior, thus affecting normal growth, development, metamorphosis, and reproduction (Blenau et al., 2012; Oboh et al., 2017). However, because invertebrates only have octopamine receptors, plant essential oils used for pest control do not pose a risk to mammals (Jankowska et al., 2018). Additionally, plant essential oils affect insect respiration, for example, by inhibiting mitochondrial membrane respiratory enzymes and controlling oxygen uptake and carbon dioxide output (Gao et al., 2020). One mechanism of plant essential oils is thought to be inhibition of the oxidative system of insects, which causes generation of reactive oxygen species and induces oxidative stress (Chen et al., 2021a). Recent transcriptome research has demonstrated that insects upregulate the expression of their detoxifying enzyme genes in response to plant secondary compounds, maintaining their ability to function normally (Sierra et al., 2021; Ding et al., 2022).

A more comprehensive understanding of agricultural pest biology has been enabled by the development of transcriptomics. The use of dsRNA to silence resistance genes in pests to improve insecticide efficiency has gradually become a hot issue. Genes associated with *E. onukii* resistance to pesticides have been reported. For example, Zhang (2019) investigated thiamethoxam resistance in *E. onukii* and discovered that numerous detoxifying genes in *E. onukii* were involved in the drug resistance process. According to Chen (2021), the P450 gene (CYP307A) was associated with imidacloprid metabolism and was crucial for nymph molting behavior. However, there are few studies on the effect of plant essential oils on the gene expression profile of *E. onukii*. Therefore, the initial stage of the present study involved identifying the differentially expressed genes through *P. cablin* essential oil stress on *E. onukii* to provide a foundation for its subsequent prevention and control.

## 2 Materials and methods

### 2.1 Plant essential oil and tested insects

The two steam-distilled essential oils used in this study (*P. cablin* and *C. camphora*) were purchased from Ji’an Zhongxiang Natural Plant Co., Ltd. (Jiangxi, China) in June, 2022. *E. onukii* were collected from Yuhuang Ancient Tea Factory in Huaxi District, Guiyang City, China (106°75′E, 26°32′N) in June, 2022. The captured insects were placed in an insect cage (75 × 75 × 75 cm) with fresh tea branches and leaves. One day later, adults exhibiting strong activity and consistent morphology were selected for use in the fumigation test.

### 2.2 Fumigation test

The fumigation activity of essential oils against *E. onukii* was tested using sealed conical flasks (Zhang et al., 2020). Plant essential oils were diluted with acetone to five concentrations: 1, 0.5, 0.25, 0.125, and 0.0625 μL mL^−1^. Twenty adult *E. onukiis* were inoculated in a 250 mL triangular flask, and fresh tea leaves were added. Using a pipette, 30 μL of treatment solution was absorbed onto the filter paper. To keep the filter paper suspended in the bottle and away from the bottle wall, it was placed in the center of the sealing film. An equivalent volume of acetone solution was then added as a blank control after sealing using the sealing film. Each treatment was repeated four times, and *E. onukii* mortality was recorded after 12, 24, and 36 h.

Data analysis was performed using SPSS (IBM, version 26.0). Fumigation data were analyzed by probit analysis. The probit-log (concentration) regression model was used to calculate lethal concentration 50% (LC50) values and 95% confidence limits.

### 2.3 Analysis of essential oil components

Gas chromatography‒mass spectrometry (GC‒MS) (HP6890/5975C, Agilent, United States) was used to analyze the essential oil components. The chromatographic column was a HP-5MS (60 m × 0.25 mm × 0.25 μm) elastic quartz capillary column. The injection volume was 0.2 µL, and the initial temperature of the chromatographic column was 70°C (2 min retention); the temperature was increased to 208°C at 3.5°C/min and then to 310°C at 6°C/min. The running time was 58.43 min; the vaporization chamber temperature was 250°C; and the carrier gas was high purity He (99.999%). The pre-column pressure was 18.43 psi, the carrier gas flow rate was 1.0 mL/min, the split ratio was 50:1, and the solvent delay time was 6 min. The ion source was electron ionization, the ion source temperature was 230°C, the quadrupole temperature was 150°C, the electron energy was 70 eV, the emission current was 34.6 μA, the multiplier voltage was 1494 V, the interface temperature was 280°C, and the mass range was 29–500 amu. The peaks in the total ion chromatogram were searched using a mass spectrometry computer data system, and the Nist17 and Wiley275 standard mass spectra were checked to determine the volatile chemical components. The relative mass fraction of each chemical component was determined using the peak area normalization method.

### 2.4 Transcriptome samples

The fumigation test method for insects was the same as that described in Section 2.2. The LC_50_ value of *P. cablin* essential oil was used as the treatment concentration. Treatment (TM1, TM2, TM3) and control groups (CK1, CK2, CK3) were set up, each with 50 individuals. After 12 h of fumigation, the adults were collected and placed under dry ice for rapid freezing and stored at −80°C. They were then transported to Shanghai Ouyi Biomedical Technology Co., Ltd. for RNA extraction, library sequencing, and data analysis.

### 2.5 RNA extraction and library construction

Total RNA was extracted from *E. onukii* using Trizol reagent (Invitrogen, United States) and DNase I (Promega, Madison, WI, United States) to remove residual DNA according to the manufacturer’s instructions. The quality of extracted RNA samples was verified by 1% agarose gel electrophoresis, and the extracted total RNA integrity (RIN value) was digitally evaluated again using an Agilent 2,100 Bioanalyzer (Agilent Technologies, Santa Clara, CA, United States). The library was constructed using the TruSeq Stranded mRNA LTSample Prep Kit (Illumina, San Diego, CA, United States) according to the manufacturer’s instructions. The constructed library was subjected to quality inspection using an Agilent 2,100 Bioanalyzer (Agilent Technologies, Santa Clara, CA, United States), and then sequenced on the Illumina HiSeq^TM^ 2,500 platform (Illumina, San Diego, CA, United States) to produce 125 or 150 bp double-ended data.

### 2.6 Data preprocessing, quality control, and assembly

Sequencing and data analysis were completed by Shanghai OE Biotech Co., Ltd. (Shanghai, China). The raw reads generated in high-throughput sequencing were fastq format sequences. Raw reads were filtered to obtain high-quality reads for subsequent analysis. First, Trimmomatic (version: 0.36) software was used for quality control and removal of joints. On this basis, low-quality and N bases were filtered out and high-quality clean reads were finally obtained (Bolger et al., 2014). We used Trinity (version: 2.4) software to *de novo* assemble the data (Grabherr et al., 2011). CD-HIT (version: 4.6) software was used to cluster and remove redundancy, and the obtained Unigene was used as a reference sequence for subsequent analysis (Li et al., 2001).

Diamond software was used to align the Unigene to Non-Redundant Protein Sequence Database (NR) (https://www.ncbi.nlm.nih.gov/), clusters of euKaryotic Orthologous Groups (KOG) (ftp://ftp.ncbi.nih.gov/pub/COG/KOG/kyva), Gene Ontology (GO) (http://www.geneontol-ogy.org/), Swiss-Prot (http://www .uniprot.org/), Evolutionary Genealogy of Genes: Non-supervised Orthologous Groups (eggNOG) (http://eggnog.embl.de/), and Kyoto Encyclopedia of Genes and Genomes (KEGG) (http://www.genome.jp/kegg/pathway.html) databases (Buchfink et al., 2015). HMMER software was used to compare the Pfam (http://pfam.xfam.org/) database for functional analysis of the Unigene (Mistry et al., 2013).

### 2.7 Differentially expressed gene screening and identification

Bowtie 2 software (version: 2.3.3.1) was used to obtain the number of reads aligned to the Unigene in each sample, and eXpress software (version: 1.5.1) was used to calculate the Unigene expression (fragments per kilobase of transcript per million mapped reads [FPKM] value) (Langmead and Salzberg, 2012; Roberts, 2013). After obtaining the counts, the protein-coding genes required filtering, and genes with mean counts >2 were retained for further analysis. DESeq2 software (version: 1.20.0) was used to standardize the counts number of each sample gene (using the BaseMean value to estimate the expression level), calculate the fold change, and for the Negative Binomial distribution test (NB) for the difference significance test (*p*-value) (Love et al., 2014). Differentially expressed genes with a *p*-value <0.05 and fold change >2 were selected, and GO and KEGG enrichment analysis were performed to determine the biological functions or pathways mainly affected by the differential Unigenes.

Unigene sequences were sequentially compared in the order of priority of NR, SwissProt, and KOG databases, and the protein with the highest score was selected to determine its coding region sequence, which was translated into the amino acid sequence according to the standard codon table to obtain the nucleic acid sequence (sequence direction 5'→3′) and amino acid sequence of this Unigene coding region. Using annotation data from seven major databases, potential detoxification-related genes were identified, and the BLASTp function of the National Center for Biotechnology Information (NCBI) was then used to confirm the protein that the candidate gene encoded to verify whether it was a detoxification-related gene.

### 2.8 Quantitative real-time‒polymerase chain reaction (qRT-PCR) real-time fluorescence quantitative verification

RNA integrity was evaluated using a NanoDrop 2000 spectrophotometer (Thermo Scientific, United States) and agarose gel electrophoresis. The RNA to be tested was reverse transcribed into cDNA using a TransScript All-in-One First-Strand cDNA Synthesis SuperMIX for qPCR (ransgen biotech, Beijing, China) kit according to the manufacturer’s instructions. Using *β*-actin (KJ476139.1) as an internal reference gene, Shanghai Ouyi Biomedical Technology Co., Ltd. designed primers for seven randomly selected genes and internal reference genes using the Primer-BLAST online program (https://www.ncbi.nlm.nih.gov/tools/primer-blast/), which were synthesized by Beijing Qingke Xinye Biotechnology Co., Ltd. The PerfectStart^TM^ Green qPCR SuperMix (transgen biotech, Beijing, China) kit was used to react on a LightCycler^®^ 480 II fluorescence quantitative PCR instrument (Roche, Swiss). The amount of gene expression was calculated using the 2^−ΔΔCt^ method (Livak and Schmittgen, 2001). The primer information of genes is shown in Table 1.

**TABLE 1:** qRT-PCR primers for differentially expressed genes of *E. onukii*.

Gene Symbol	Forward primer (5->3)	Reverse primer (5->3)
β-actin	GAT​CTG​GCA​TCA​CAC​CTT​CTA	TGA​GTC​ATC​TTC​TCC​CTG​TT
TRINITY_DN31847_c0_g1_i1_2	TTC​CTG​GCT​GTA​GAG​TGT​GA	TAG​TGT​GCG​CAA​ACT​CTT​C
TRINITY_DN37711_c2_g1_i3_1	GTC​ACA​TAT​TTG​CGG​AAC​TG	GTT​AGA​TTT​CCC​TTG​ACC​ATT​G
TRINITY_DN31516_c0_g1_i2_2	CTAGCCACCACTTACGG	CAT​TTG​CTG​GTC​GTA​AGG​A
TRINITY_DN33042_c0_g1_i2_1	CAT​TCT​CAT​ATC​AGA​CTG​TCC​T	AGA​TGC​TGG​CAC​ATA​TAC​C
TRINITY_DN37682_c1_g2_i1_1	TTTACAATGGCGTTGGGC	GGA​GAG​AAG​AGA​GAG​AAG​CTA
TRINITY_DN32309_c0_g1_i1_1	GCGACATTGCCACTTGTA	CTT​GTC​TTT​TCC​AAC​CTG​AGA​T
TRINITY_DN24376_c0_g1_i1_2	CTG​GTA​GTG​AAC​GTG​ACA​GA	GTACTTGGTGCTGTCCTT

## 3 Results

### 3.1 Fumigation activity

Table 2 displays the fumigation toxicity of essential oils from *P. cablin* and *C. camphora* to *E. onukii*. The 12-, 24-, and 36-h LC_50_ values of *E. onukii* treated with *P. cablin* essential oil were 1.041, 0.659, and 0.474 μL mL^−1^, respectively (Table 2; Supplementary Table S13). The 12-, 24-, and 36-h LC_50_ values of *E. onukii* treated with *C. camphora* essential oil were 2.21, 1.581, and 1.204 μL mL^−1^, respectively. The LC_50_ value decreased gradually with the increase in exposure time. The 12-h LC_50_ value of *P. cablin* oil was used as the subsequent transcriptome treatment concentration.

**TABLE 2:** Toxicity of two essential oils to the fumigation and killing of *E. onukii*.

Essential oil types	Time hour	Toxicity regression equation	LC_50_ μL·mL^-1^	LC_50_ (95%CL) μL·mL^-1^	r	X^2^
*P. cablin* essential oil	12	Y = −1.193 + 1.146X	1.041	0.872–1.331	0.985	3.205
	24	Y = −0.786 + 1.193X	0.659	0.408–1.505	0.947	7.673
	36	Y = −0.607 + 1.28X	0.474	0.263–0.789	0.955	5.672
*C. camphora* essential oil	12	Y = −1.772 + 0.779X	2.21	1.569–4.442	0.963	2.539
	24	Y = −1.541 + 0.974X	1.581	1.244–2.336	0.977	3.205
	36	Y = −1.377 + 1.144X	1.204	1–1.573	0.982	4.341

A total of 26 compounds were identified in *P. cablin* essential oil. Table 3 shows that *patchouli* alcohol, *δ*-guaiene, *α*-guaiene, cembrene, *α*-patchoulene, aciphyllene, caryophyllene, and *β*-patchoulene were the main components of *P. cablin* essential oil, accounting for 85.124% of the total essential oils (Supplementary Figure S1B). The highest content was that of *patchouli* alcohol (30.811%), and the lowest was that of D-ramie (0.012%).

**TABLE 3:** Chemical composition of *P. cablin* essential oil.

Retention time(min)	Compounds	Molecular formula	Molecular weight	Peak area	Percentage content%
38.682	Patchouli alcohol	C_15_H_26_O	222	378,793,093	30.811
33.076	δ-Guaiene	C_15_H_24_	204	210,044,850	17.085
30.597	α-Guaiene	C_15_H_24_	204	168,566,963	13.711
31.021	Seychellene	C_15_H_24_	204	89,160,586	7.252
31.484	α-Patchoulene	C_15_H_24_	204	62,634,081	5.095
32.72	Aciphyllene	C_15_H_24_	204	51,608,675	4.198
29.984	Caryophyllene	C_15_H_24_	204	44,099,267	3.587
28.601	β-patchoulene	C_15_H_24_	204	41,615,386	3.385
38.431	1,4-Dimethyl-7-(prop-1-en-2-yl)-decahydroazulen-4-ol	C_15_H_26_O	222	24,717,273	2.01
31.589	(1R,3aS,8aS)-7-Isopropyl-1,4-dimethyl-1,2,3,3a,6,8a-hexahydroazulene	C_15_H_24_	204	24,538,709	1.996
28.751	β-elemene	C_15_H_24_	204	17,957,825	1.461
39.838	4-Hydroxy-6-methyl-3-(4-methylpentanoyl)-2H-pyran-2-one	C_12_H_16_O_4_	224	15,638,562	1.272
31.691	Patchoulene	C_15_H_24_	204	11,921,854	0.97
29.728	(1S,1aS,1bR,4S,5S,5aS,6aR)-1a,1b,4,5a-Tetramethyldecahydro-1,5-methanocyclopropa[a]indene	C_15_H_24_	204	9,246,159	0.752
31.246	Humulene	C_15_H_24_	204	8,406,340	0.684
35.767	Caryophyllene oxide	C_15_H_24_O	220	5,153,823	0.419
33.514	(2S,4aR,8aR)-4a,8-Dimethyl-2-(prop-1-en-2-yl)-1,2,3,4,4a,5,6,8a-octahydronaphthalene	C_15_H_24_	204	3,491,916	0.284
26.565	δ-Elemene	C_15_H_24_	204	2,455,778	0.2
39.695	(3S,5R,8S)-3,8-Dimethyl-5-(prop-1-en-2-yl)-2,3,5,6,7,8-hexahydroazulen-1(4H)-one	C_15_H_22_O	218	1,914,371	0.156
12.599	β-Pinene	C_10_H_16_	136	1,608,667	0.131
32.062	γ-Gurjunene	C_15_H_24_	204	918,237	0.075
32.243	Pentadecane	C_15_H_32_	212	682,481	0.056
11.071	α-Pinene	C_10_H_16_	136	675,741	0.055
27.392	Eugenol	C_10_H_12_O_2_	164	290,826	0.024
29.449	Cis-caryophyllene	C_15_H_24_	204	237,248	0.019
14.411	D-Limonene	C_10_H_16_	136	151,037	0.012

### 3.2 De novo assembly

**FIGURE 1:**
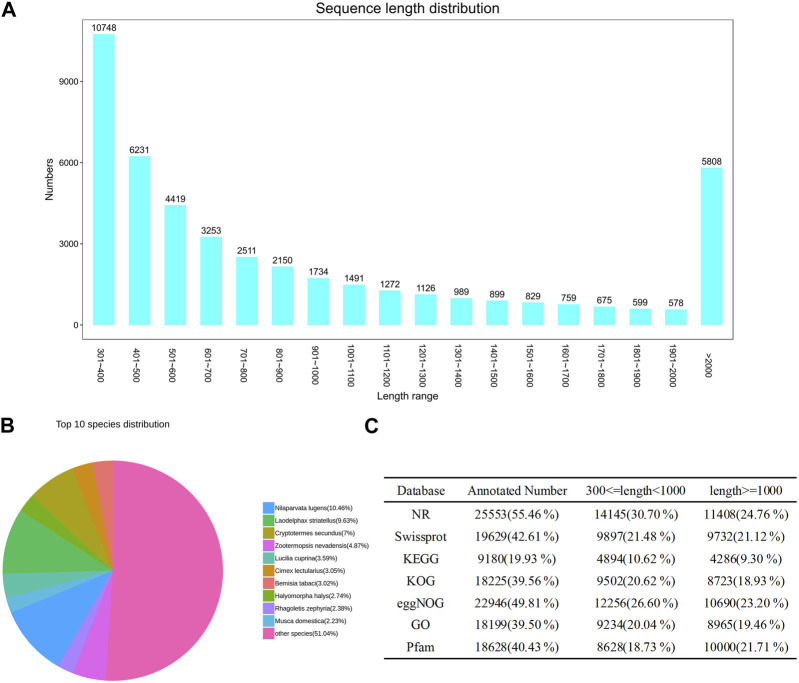
**(A)** Unigene length distribution diagram showing the length of the assembled transcripts generated in the *de novo* assembly using Trinity after combining all RNA-seq replicates. **(B)** Annotated top 10 species distribution in NR database, fan shapes of different colors indicate the proportion of annotated species. **(C)** Number of unigenes annotated to different databases (NR, Swissprot, KEGG, KOG, eggNOG, GO, Pfam).

After quality control, we assembled the transcriptome data (Supplementary Figure S1A). The transcript sequence was obtained using Trinity software (version 2.4), and the longest sequence was selected as the Unigene. The Unigene was obtained by clustering and removing redundancy using CD-HIT software (version 4.6). Finally, 46,071 Unigenes were obtained, with a total length of 48,202,476 bp and a length distribution ranging from 301 to 23,439 bp (Supplementary Date Sheet S1). Among the obtained Unigenes, 16,979 had a length from 300 to 500 bp, accounting for 36.85% of the total Unigenes. A total of 14,067 were located from 500 to 1,000 bp, accounting for 30.53% of the total Unigenes. There were 9,217 Unigenes located from 1,000 to 2000 bp, accounting for 20% of the total Unigenes. The N50 length of Unigenes was 1,554 bp, which was greater than the average length (1,046.27 bp), indicating high integrity of the gene samples (Figure 1A; Supplementary Table S9A).

### 3.3 Functional annotation of all unigenes

**FIGURE 2:**
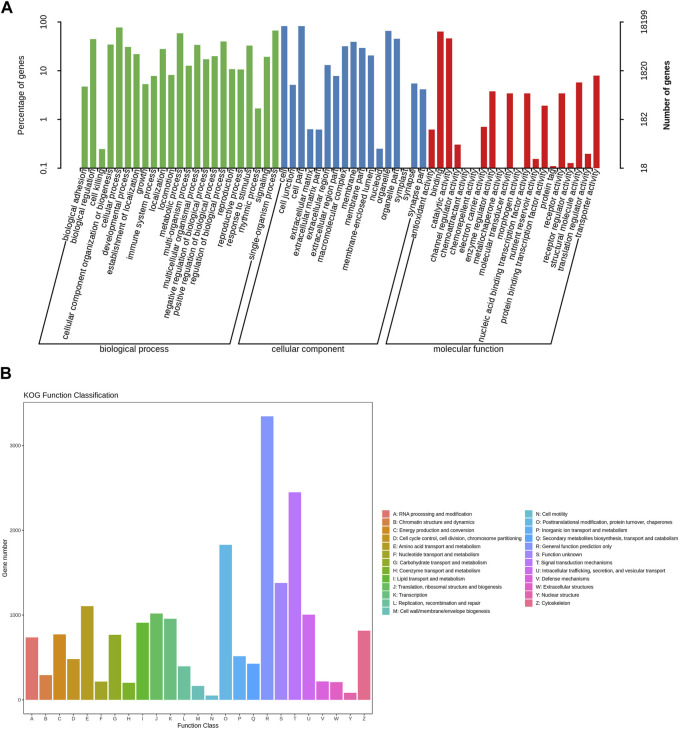
The functional annotation of assembled unigenes of *E. onukii*. **(A)** GO classifications of assembled unigenes. **(B)** KOG categories of assembled unigenes.

We annotated the obtained Unigenes in NR, Swissprot, KEGG, KOG, eggNOG, GO, and Pfam, from which the numbers of annotated genes were 25,553 (55.46%), 19,629 (42.61%), 9,180 (19.93%), 18,225 (39.56%), 22,946 (49.81%), 18,199 (39.50%), and 18,628 (40.43%), respectively (Figure 1C; Supplementary Table S8). There were 6,421 Unigenes annotated in all seven databases, accounting for 13.94% of the total Unigenes. The NR database had the highest annotation rate, with 25,553 Unigenes annotated, accounting for 55.46% of the total; of these, 14,145 (30.70%) were between 300 and 1,000 bases, and 11,408 (24.76%) were >1,000 bases. The gene sequences of known species in the NR database were compared to the Unigene constructed for *E. onukii*. *Nilaparvata lugens* (10.46%), *Laodelphax striatellus* (9.63%), and *Cryptotermes secundus* (7%) had the highest alignment rates; i.e., 2,672, 2,462, and 1,789 genes were aligned, respectively (Figure 1B; Supplementary Table S9B). The genes annotated into the GO database could be divided into three categories: biological process, cell component, and molecular function, and could be further divided into 60 subcategories according to their specific functions (Figure 2A; Supplementary Table S10A); the terms “cellular process,” “cell,” and “cell part” were the most dominant, respectively. The assembled Unigenes were annotated in the KOG database and the annotated genes could be divided into 25 functional categories (Figure 2B; Supplementary Table S10B). The most annotated genes were general function prediction only (3,345), followed by signal transduction mechanisms (2,448) and posttranslational modification, protein turnover, and chaperones (1,829).

### 3.4 Screening and functional analyses of differentially expressed genes

**FIGURE 3:**
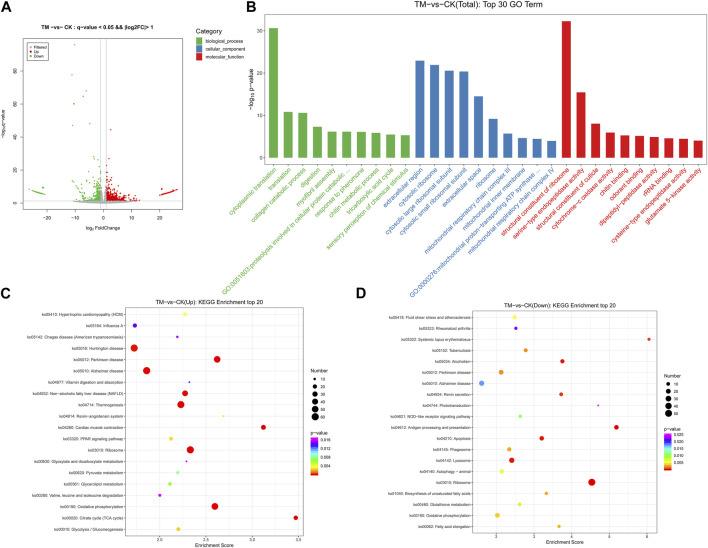
**(A)** Volcano map of differentially expressed genes (DEGs) of *E. onukii* after exposure of adult to the LC_50_ value of *P. cablin* essential oil. **(B)** Gene Ontology (GO) top 30 enriched terms of differentially expressed genes (DEGs) of *E. onukii* after exposure of adult to the LC_50_ value of *P. cablin* essential oil. **(C)** Bubble map of the upregulated gene KEGG enrichment pathway and **(D)** the downregulated gene KEGG enrichment pathway of *E. onukii* after exposure of adult to the LC_50_ value of *P. cablin* essential oil. Pathway significance is shown together with *p*-value (color), rich factor (vertical ordinate), and a number of involved genes (size of circles).

After obtaining the FPKM values of all genes, differentially expressed genes were screened (Supplementary Tables S5–S7). A total of 2,309 differentially expressed genes were found between the control and treatment groups, of which 1,597 showed upregulation and 712 showed downregulation (Supplementary Table S2). The differential expression volcano map is shown in Figure 3A; Supplementary Table S11A. The greater the difference multiple and the higher the -log10 *p*-value, the stronger the significance of the gene difference and the further the abscissa was from the zero scale.

From the 2,309 differentially expressed genes, a total of 1,211 genes were annotated to the GO functional database, and the top 10 significantly enriched items in three categories were selected (Figure 3B; Supplementary Table S11B). The most significantly enriched biological processes were cytoplasmic translation, translation, and the collagen catabolic process, with 62, 64, and 34 genes, respectively, accounting for 5.12, 5.28, and 2.81%, respectively, of the total. The extracellular region, cytosolic ribosome, and cytosolic large ribosomal subunit were the three most significantly enriched cell components, with 172 (14.2%), 42 (3.47%), and 52 (4.29%) genes, respectively. Among the molecular functions, the structural constituent of ribosome, serine-type endopeptidase activity, and structural constituent of cuticle were the most significantly enriched, with 120 (9.91%), 82 (6.77%), and 23 (1.9%) genes, respectively.

KEGG enrichment analysis showed that a total of 488 differentially expressed genes were categorized into 292 pathways (Supplementary Table S11C). Figure 3C presents the most significant enrichment of the top 20 pathways. Oxidative phosphorylation, Parkinson disease, ribosome, thermogenesis, cardiac muscle contraction, the tricarboxylic acid cycle, Alzheimer disease, non-alcoholic fatty liver disease, and other pathways, were significantly enriched, and the numbers of differentially expressed genes were 52, 50, 59, 57, 26, 21, 60, and 37, respectively. A total of 217 differentially expressed genes were classified into 199 pathways, according to KEGG enrichment analysis of downregulated genes. Ribosome, antigen processing and presentation, apoptosis, alcoholism, lysosome, renin secretion, systemic lupus erythematosus, and tuberculosis had 51, 17, 16, 132, 24, 10, 6, and 11 differential genes, respectively (Figure 3D; Supplementary Table S11D).

### 3.5 qRT-PCR verification

**FIGURE 4:**
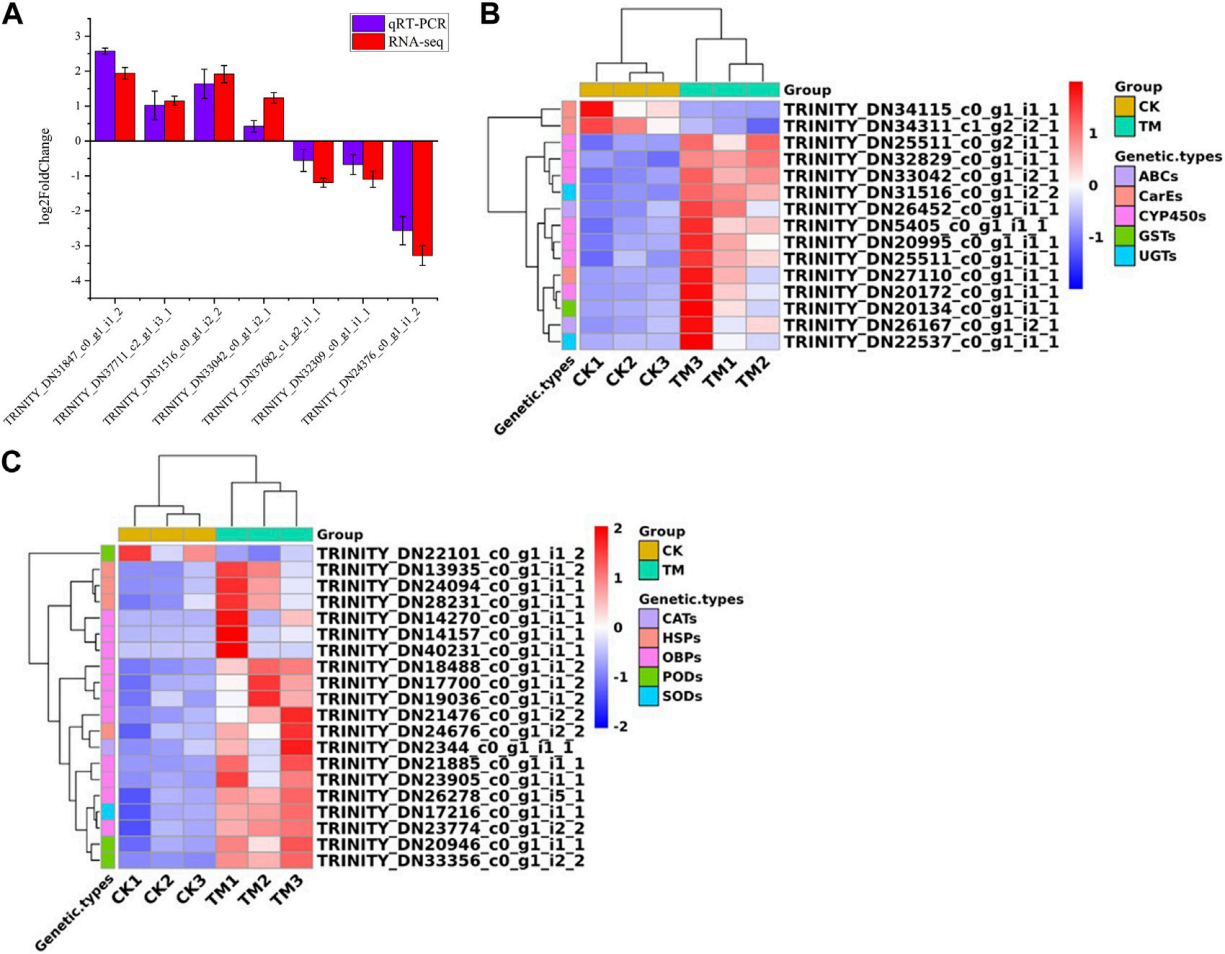
**(A)** Quantitative Real-time PCR validation of transcriptome sequencing results. **(B)** Differentially expressed genes related to detoxification among differential genes. **(C)** Differentially expressed genes related to redox and stress resistance. The gene expression (mean ± SD) quantified as a relative fold change was carried outusing the 2^−ΔΔCT^ method.

To verify the accuracy of RNA-seq sequencing results, we used β-actin as an internal reference gene, and randomly selected seven genes among the differentially expressed genes for verification (Supplementary Figures S2A–P). Figure 4A shows that the qRT-PCR sequencing results were consistent with the RNA-seq results, indicating that the results of the above transcriptome sequencing were accurate and reliable (Supplementary Table S12).

### 3.6 Analysis of detoxification-related genes in differentially expressed genes

Through identification and screening of differential genes, we found 14 detoxification genes (Figure 4B; Supplementary Table S3). Seven CYP450 genes were identified, all of which showed upregulation and had log2FoldChange values ranging from 1.96 to 4.37. Two carboxylesterase (CarE) genes showed downregulation; whereas, one GST gene, two ABC genes, the UGT gene, and one CarE gene showed upregulation.

Among the differentially expressed genes, we identified redox genes, including one upregulated superoxide dismutase (SOD) gene; one upregulated catalase (CAT) gene; two upregulated peroxidase (POD) genes; and one downregulated POD gene (Figure 4C; Supplementary Table S4). Furthermore, 11 OBP genes that may be involved in drug tolerance, and four heat shock protein (HSP) genes associated with insect stress resistance, showed upregulation.

## 4 Discussion

*P*. *cablin* essential oil and its related compounds have been demonstrated to have some insecticidal efficacy against a variety of pests in numerous studies. Depending on where it is obtained from, *P. cablin* essential oil may be categorized as transitional, *patchouli* alcohol, or pogostone type (Luo et al., 2003; Hu et al., 2006). According to the GC‒MS results, the first six main components of the tested essential oil were *patchouli* alcohol (30.811%), *δ*-guaiene (17.085%), *α*-guaiene (13.711%), caryophyllene (7.252%), *α*-patchoulene (5.095%), and pogostone (1.272%); hence, the essential oil was *patchouli* alcohol type. The most commonly reported bioactive substance found in *P. cablin* is pogostone. Zeng et al. (2006) found that *P. cablin* essential oil and its compound pogostone had an anti-feedant effect on *Pieris rapae* and *Plutella xylostella* larvae and certain contact activity against *P. rapae*. In addition, *patchouli* alcohol has also been reported to have strong insecticidal activity against *Aedes aegypti* (Muturi et al., 2019).

Essential oil from *C. camphora* and its compounds have insecticidal properties against a variety of insects. The first seven primary components of oil from *C. camphora* were determined by GC‒MS to be (+)-2-*camphor* (25.817%), safrole (13.597%), eucalyptol (11.269%), linalool (6.343%), nerolidol (5.852%), D-limonene (5.359%), and *α*-terpineol (4.618%). *Cinnamomum camphora* essential oil and its main component eucalyptol are effective against *Anopheles stephensi* larva and *P. xylostella* (Xu et al., 2020; Huang et al., 2021). Linalool is toxic to *Aphis gossypii* and *Anticarsia gemmatalis* (Jiang et al., 2016; Vicenco et al., 2021). Safrole is toxic to *Sitophilus zeamais* and *A. aegypti* (Carmen et al., 2015; Liu et al., 2015). D-limonene has strong fumigation, repellent, and ovicidal effects on *Callosobruchus maculatus*, *Tribolium castaneum*, *Lasioderma serricorne*, and *Loposcelis bostrychophila* (Qi et al., 2021; Rodrigues et al., 2022). The fumigation activity of essential oils from *P. cablin* and *C. camphora* against *E. onukii* was evaluated using sealed conical flasks, but tea green leafhoppers damage the tea in the open field. Therefore, this should be considered a limitation in the study and future research would address contact toxicity assays to validate our results both in the laboratory and in the field.

In our study, the 36-h semi-lethal concentrations of *P. cablin* and *C. camphora* essential oils against *E. onukii* were 0.474 and 1.204 μL mL^−1^, respectively. The fumigation toxicity exhibited certain time- and concentration-dependent effects. With increases in time and concentration, the insecticidal activity was further improved, which was consistent with the fumigation results of *P. cablin* essential oil against *Tetranychus cinnabarinus* (Cheng et al., 2020). The fact that many of the compounds in the essential oil exhibit a wide range of biological activities, including contact killing, repulsiveness, growth and development inhibition, and fumigation against other insects, suggests that the essential oil may also be effective against *E. onukii*.

It is generally believed that detoxification of exogenous substances by insects is mainly divided into three stages. Stage I mainly involves oxidation, reduction, and hydrolysis of exogenous substances involved in P450 enzymes and esterases, which makes toxic molecules introduce polar groups (Schama et al., 2016). Cytochrome P450 monooxygenases (P450s) catalyze multiple oxidative transformations of endogenous and exogenous substrates (Li et al., 2007; Nelson, 2017), which plays an important role in plant‒host interactions and metabolism of different insecticides (Philippou et al., 2010; Alptekin et al., 2016). Insecticides and plant secondary compounds are highly metabolized by several CYP450 family genes (Li et al., 2004; Sasabe et al., 2004; Wang et al., 2017). In our study, a total of seven CYP450 genes were upregulated in the essential oil treatment group, indicating that CYP450 genes may have been involved in the detoxification and metabolism of *P. cablin* essential oil. One of the most common varieties of esterases is CarE, which performs a wide range of physiological functions in organisms, participating in the metabolism of several endogenous compounds (including hormones, pheromones, and neurotransmitters) as well as the detoxification of various exogenous substances (Wei et al., 2019; Hilliou et al., 2021). CarEs play a key role in the defense of various plant secondary metabolites related to plants and insecticides (Oakeshott et al., 2005). In the present study, two CarE genes were downregulated, indicating that *P. cablin* essential oil may have inhibited the role of CarE in phase I detoxification metabolism by reducing expression of CarE genes in *E. onukii*. This is consistent with the reported inhibition of plant essential oil on CarE activity of *S. zeamais*, *P. xylostella* larvae, and *Haemaphysalis longicornis* (Liao et al., 2016; Qiao et al., 2021).

The process of binding reaction products in the second stage, which is mostly performed by GSTs and UGTs, causes the reaction products from stage I to join with a number of endogenous substances (such as glutathione [GSH] and glucuronic acid) to generate water-soluble molecules (Glisic et al., 2015; Lushchak et al., 2018). GSTs are extremely varied detoxifying phase II enzymes. In addition to their roles in intracellular transport and oxidative stress reduction, GSTs facilitate excretion of harmful substances out of the cell by increasing the binding of reduced GSH to toxic substances (Lushchak, 2012; Zhu and Luttrell, 2015). In our study, one GST gene was upregulated and may have been involved in the process of catalyzing the binding of GSH to toxic substances, thereby reducing the toxicity of plant essential oils to the insects (Liao et al., 2016; Gao et al., 2020). Many different organisms contain UDP glucuronosyltransferase families (UGTs), the mechanisms of action of which resemble those of GSTs. Two UGT genes were upregulated in our study, suggesting that *E. onukii* may hasten the hydrolysis rate of the compound by highly expressing UGTs.

ATP-binding cassette transporters are crucial in stage III, which primarily delivers the reaction products from stage II to the extracellular area (Vieira-Brock et al., 2013). ABC transporters either isolate harmful molecules into tissues that are not metabolically active, rendering them inert, or move some toxic compounds away from their targets (Medina et al., 2002; Gott et al., 2017). The detoxification mediated by ABC transporters in many insects has attracted attention (You et al., 2013; Mastrantonio et al., 2017). Based on the aforementioned circumstance, we hypothesize that the ABC transporter in *E. onukii* may have accelerated the excretion efficiency of essential oils and indirectly participated in the detoxification process.

One of the mechanisms of insecticides is thought to be their ability to cause oxidative stress in insects (Akbar et al., 2012; Lazarevic et al., 2020; Czerniewicz and Chrzanowski, 2021). In our study, we found significant enrichment of superoxide dismutase activity (GO: 0004784), response to oxidative stress (GO: 0006979), oxidation-reduction process (GO: 0055114) and response to ethanol (GO: 0045471) pathways. Two SOD genes, one CAT gene, and two POD genes were upregulated, and one POD gene was downregulated. Other insects also exhibit an increase in the transcriptional expression level of antioxidant enzymes induced by toxic compounds; two SOD genes (MnSOD and ecCuZnSOD1) in *Oxya chinensis* had higher expression levels after exposure to the insecticide malathion; when these two genes were knocked out, the level of reactive oxygen species in the body increased considerably (Wu et al., 2017). Therefore, we infer that after fumigation with *P. cablin* essential oil, *E. onukii* may have produced a certain amount of reactive oxygen species, which increased the transcription level of antioxidant enzymes in the body so as to eliminate the excess reactive oxygen species in the cells to maintain the oxidative balance.

Nine OBPs genes were found in the two enriched pathways of odorant binding (GO: 0005549) and sensory perception of chemical stimulus (GO: 0007606). OBPs are extremely small water-soluble secretory proteins that are found in the lymph of insect olfactory receptors. They play an important role in the selective binding and transportation of fat-soluble odor molecules and have other functions such as anti-inflammatory effects, regeneration and development, visual pigment carrier, and nutritional effects (Leal, 2013; Pelosi et al., 2014; Pelosi et al., 2018). Recent studies have shown that the high expression of these genes is related to the drug resistance of various insects such as *Diaphorina citri*, *N. lugens*, *Culex quinquefasciatus*, and *T*. *castaneum* (Liu et al., 2020; Gao et al., 2021; Shen et al., 2022; Zhang et al., 2022). Expressions of nine OBPs were upregulated in this study, and we theorized that this increased the tolerance of *E. onukii* to essential oil. Insect HSPs play an important role under heat stress, cold stress, and insecticide treatment (Koo et al., 2015; Dumas et al., 2019). We found that four HSPs were upregulated between the differential genes. Interestingly, similar phenomena also occur when other insects are exposed to insecticide stress (Dong et al., 2022). The HSP90, sHSP, and HSP70 gene families of *Anopheles sinensis* are considered to be involved in pyrethroid resistance (Si et al., 2019). We hypothesized that the upregulated HSP may have enhanced stress resistance in *E. onukii*.

GO enrichment analysis showed that items related to mitochondrial respiration were significantly enriched (*p* < 0.05), including cytochrome c oxidase activity (GO: 0004129), mitochondrial respiratory chain complex III (GO: 0005750), mitochondrial electron transport, ubiquinol to cytochrome c (GO: 0006122), mitochondrial respiratory chain complex IV (GO: 0005751), and NADH dehydrogenase (ubiquinone) activity (GO: 0008137). The genes involved in the above items were significantly upregulated, indicating that plant essential oils affected respiration. In addition, in the KEGG pathway, we found that the transcripts in the respiration-related oxidative phosphorylation (ko00190) glycolysis/gluconeogenesis (ko00010) citrate cycle (tricarboxylic acid cycle) (ko00020) pathway were significantly upregulated, indicating enhanced respiration after essential oil exposure. Most insecticides have some effect in inhibiting insect respiration, for example, pyrethroids, which stop the oxidative phosphorylation chain and lower the respiratory rate in *Anopheles gambiae* (Ingham et al., 2021). It appeared that inhibiting *E. onukii* respiration was not the target of the essential oil in the present study. Excessive energy used for respiration produces may be used to achieve detoxification (Mu et al., 2005).

## 5 Conclusion

*P. cablin* essential oil demonstrated strong insecticidal action, according to the examination of the constituent parts of two essential oils. *P. cablin* essential oil-stressed transcriptome sequencing of *E. onukii* revealed 2,309 differentially expressed genes between the treatment and control groups. These genes belonged to five gene families involved in detoxification, as well as redox, HSP, and OBP genes. The identification of these genes offers a theoretical framework for further investigation and development of effective, low-toxicity insecticides for managing *E. onukii*. In addition, due to the complex composition of the essential oils used in this study, the toxicity of the single compound of the essential oil and the mixture of various compounds can be further compared in future studies to determine the main compounds that exert biological activity and the mixing modes to achieve the optimal control effect.

## Data Availability

The datasets presented in this study can be found in online repositories. The names of the repository/repositories and accession number(s) can be found below: https://www.ncbi.nlm.nih.gov/bioproject/PRJNA974347.
