# Supplementary figures and images for: Fumigant activity and transcriptomic analysis of two plant essential oils against the tea green leafhopper, Empoasca onukii Matsuda
Source: Front Physiol. 2023 Sep 19;14:1217608. doi: 10.3389/fphys.2023.1217608 (PMC10546945; doi:10.3389/fphys.2023.1217608)

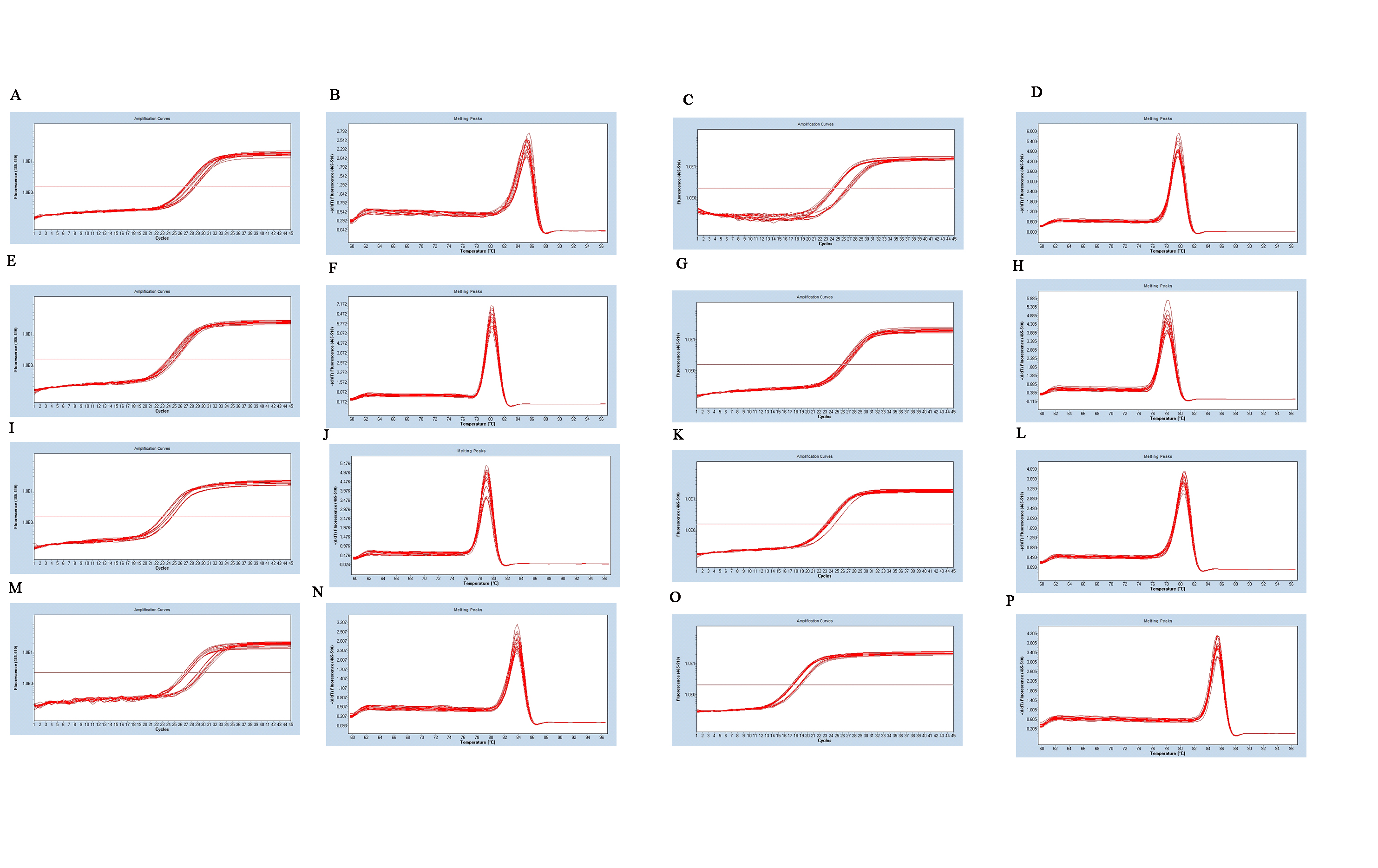

Supplement: Supplementary file 2 [file Image2.jpg]

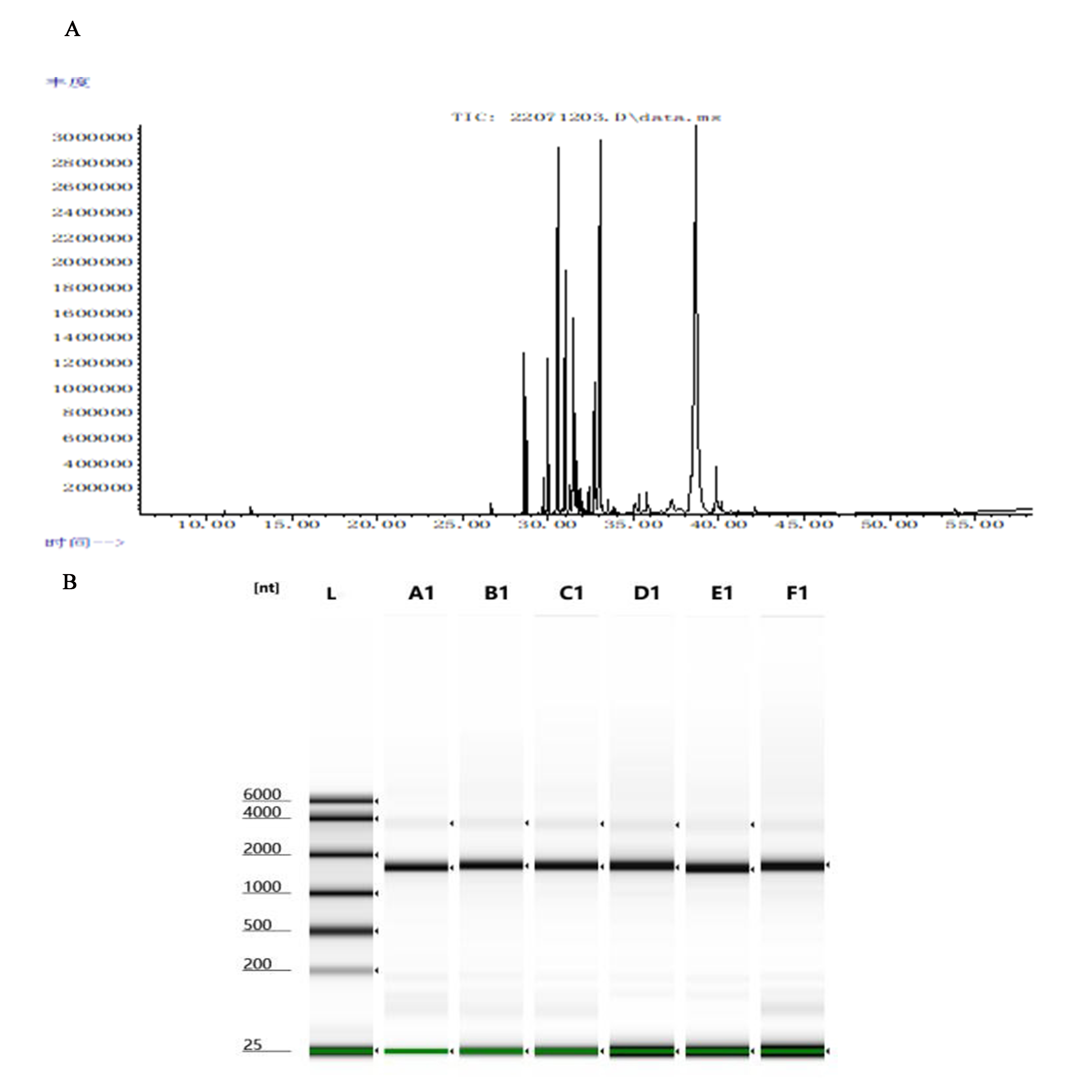

Supplement: Supplementary file 16 [file Image1.jpg]
